# An Unusual Presentation of Human Parotid Filariasis

**DOI:** 10.3390/tropicalmed10120340

**Published:** 2025-12-01

**Authors:** Tanaya Siripoon, Suppachok Kirdlarp, Polrat Wilairatana, Viravarn Luvira, Prakaykaew Charunwatthana, Parnpen Viriyavejakul, Paron Dekumyoy

**Affiliations:** 1 Department of Clinical Tropical Medicine, Faculty of Tropical Medicine, Mahidol University, Bangkok 10400, Thailand; tanaya.sir@mahidol.ac.th (T.S.); viravarn.luv@mahidol.ac.th (V.L.); prakaykaew.cha@mahidol.ac.th (P.C.); 2 Ramathibodi Medical School, Chakrinaruebodindra Medical Institute, Faculty of Medicine, Ramathibodi Hospital, Mahidol University, Samut Prakan 10540, Thailand; suppachok.kir@mahidol.ac.th; 3 Mahidol Oxford Tropical Medicine Research Unit, Faculty of Tropical Medicine, Mahidol University, Bangkok 10400, Thailand; 4 Department of Tropical Pathology, Faculty of Tropical Medicine, Mahidol University, Bangkok 10400, Thailand; parnpen.vir@mahidol.ac.th; 5 Department of Helminthology, Faculty of Tropical Medicine, Mahidol University, Bangkok 10400, Thailand; paron.dek@mahidol.edu

**Keywords:** filariasis, parotid filariasis, *Wuchereria bancrofti*

## Abstract

Human filariasis caused by *Wuchereria bancrofti* and *Brugia malayi* continues to circulate within Northern and Central Thailand and Southern Thailand, respectively. Major clinical presentations comprise lymphedema of extremities, hydrocele, funiculitis, orchitis, and tropical pulmonary eosinophilia. Microfilaria in other organs is rare. We report an unusual case of a 48-year-old woman from Southern Thailand with parotid filariasis presenting with chronic parotid gland enlargement. *Wuchereria bancrofti* microfilaria was observed within cytologic smear samples from the swollen left parotid gland and subsequently confirmed via a positive filaria immunoblot. The patient’s condition was successfully resolved through administration of a triple regimen consisting of three antiparasitic medications.

## 1. Introduction

Human filariasis remains classified as a neglected tropical disease. Instigated by the mosquito-borne filarial nematodes, *Wuchereria bancrofti*, *Brugia malayi*, and *Brugia timori*, this infection persists as a public health burden with an estimated 51 million people infected as of 2018, in addition to the 657 million people from 39 countries requiring preventive chemotherapy [1]. The World Health Organization (WHO) advocates the implementation of surveillance programs within all endemic countries by 2030 as a means to maintain 80% disease elimination; 35 countries have yet to achieve this benchmark in 2024 [2]. Lymphatic filariasis is endemic in South Asia, sub-Saharan Africa, the Pacific regions, and some parts of South America. In Thailand, only *Wuchereria bancrofti* and *Brugia malayi* are endemic to Northern and Central Thailand and Southern Thailand, respectively. The geographic distribution of *Wuchereria bancrofti* consists of Mae Hong Son, Chiangmai, Lumphoon, Tak, Kanchanaburi, Ratchaburi, and Ranong provinces [3]. Meanwhile, *Brugia malayi* is mainly found in the southern provinces, especially Narathiwat, Surat Thani, Nakhon Si Thammarat, Phatthalung, and Pattani [4,5]. However, both species are infrequently reported in other provinces within the mentioned regions of Thailand [6].

An infected individual may present with asymptomatic microfilaremia, acute adenolymphangitis, or acute filarial fever without lymphatic inflammation. Repeated episodes of acute filarial infection progress to a chronic lymphatic disease state with pathological presentations, including but not limited to lymphedema of the legs, arms, breasts, and male genitalia. Common complications include secondary bacterial infections and overlying sclerotic skin alterations. Due to lymphatic system impairment, infected patients can experience pain and develop dysfunctionality of the affected organs, causing mental and physical debilitation, financial loss, and social stigma. The estimated cost of the economic burden of chronic filariasis was US $115 per case, with a total economic burden of US $5.8 billion annually prior to the Global Programme to Eliminate Lymphatic Filariasis (GPELF) initiation [7,8]. We report an atypical case of human filariasis from Southern Thailand and review other unusual presentations of the disease, highlighting the diagnostic and control challenges in Thailand, an upper-middle-income country, to enhance early clinical recognition and diagnosis, treatment, and prevention education for filariasis.

## 2. Case Presentation

**Figure 1:**
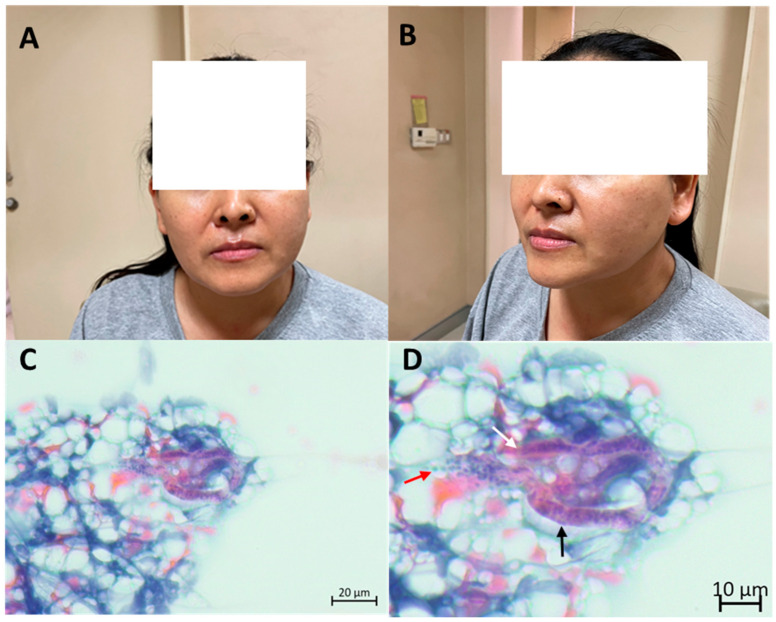
Patient’s presentation and fine-needle aspiration cytological findings, which confirm filariasis: left parotid gland enlargement as observed from the front view (**A**) and side view (**B**), cytological examination depicting suspected microfilaria in low magnification (**C**), and high magnification (**D**) exhibiting nuclei packed densely in a column running along the length of the organism (black arrow), blunt anterior end (white arrow) and nuclei which extends caudally (red arrow).

A 48-year-old Thai woman who resides in Surat Thani Province, upper Southern Thailand, presented with a 6-month history of left-sided facial swelling. She was afebrile and denied any additional symptoms or recent travel. She owns a dog, and her residence was located near a rubber forest, where she experienced frequent mosquito bites. Physical examination revealed a 4 × 5 cm non-tender soft enlargement of the left parotid gland (Figure 1A,B). Other findings were within normal limits. Laboratory results showed a normal white blood cell count of 7430 cells/mm^3^ with eosinophilia (absolute eosinophil count 891 cells/mm^3^). Other laboratory findings were within normal limits.

The initial differential diagnoses included parotid tumor and parotid tuberculosis. She underwent a CT scan of the chest. The CT scan revealed a 2.2 × 1.2 cm well-defined oval-shaped non-enhanced lesion in the prevascular space, which was later found to be a thymoma by surgery. Fine-needle aspiration cytologic smear of the left parotid gland revealed lymphoid cells of various maturation and a few polymorphonuclear cells. Large cells mimicking acinar cells and fibrovascular strands were included. One curved worm was detected (Figure 1C,D). The blunt anterior end was vaguely discernible. The sheath was not clear. Discrete nuclei were seen within the interior of the worm and extended caudally. The microscopic findings were highly suggestive of *Wuchereria bancrofti* microfilaria based on morphology. No malignancy was detected. The Knott’s concentration test, utilized to analyze nighttime blood samples, which were sent at 10 p.m. for 3 days, revealed negative results. An in-house IgG-immunoblot for filaria incorporated with an IgG4-rapid test kit produced positive results. Parotid filariasis was confirmed by immunoblot and fine-needle aspiration cytologic results. Blood smear was not performed. The patient was treated with a triple regimen of diethylcarbamazine 6 mg/kg, ivermectin 200 mcg/kg, and albendazole 400 mg single dose, and the specified regimen was repeated in the following year. The parotid gland swelling substantially decreased in volume, and the patient continued to appear well throughout a year of follow-ups. Both immunoblot and filariasis rapid test kit results were negative 1-year post-treatment.

## 3. Discussion

Human filariasis remains a significant neglected tropical disease, causing physical disability, psychological, and economic burden. Typical presentations, such as acute adenolymphangitis, acute fever with lymphadenitis, tropical pulmonary eosinophilia, hydrocele, and elephantiasis, usually lead to an accurate diagnosis and treatment. However, unusual presentations may hinder diagnosis and cause physical and psychological consequences.

The presence of microfilaria within unusual sites is an incidental finding with a limited number of cases reported due to underrecognition. Previously reported detection sites include the cervicovaginal area, thyroid, pericardial effusion, lungs, breast cyst or mass, metastatic lymph nodes or lymph nodes at various sites, ascites, scrotal fluid, within abscess, and bone marrow [9,10,11,12,13,14]. Most cases were incidental findings reported in India, whereby filariasis was not initially suspected. The exact pathophysiological mechanism remains unclear, although a feasible theory entails how microfilaremia causes lymphatic and vascular obstruction, which subsequently leads to microfilaria extravasation in various organs [14].

Challenges in labeling microfilaria as a differential diagnosis or official diagnosis inherently exist due to potential presentations in atypical locations and conditions that mimic other organ-specific pathologies. However, microfilaria will likely be present upon careful examination of pathological sites [15]. Species identification mainly relies on morphological features, with fine-needle aspiration cytological smears as the main diagnostic method. Adult stage worm specimens can be further identified through integrating histopathological evaluations and molecular analysis [16]. A summary of previously reported microfilaria from cytologic smears is shown in Table 1.

Decades ago, Southern Thailand was considered an endemic area for lymphatic filariasis, especially *B. malayi*. Upon successful implementation of disease elimination initiatives comprising mass drug administration (MDA) and surveys (both Stop-MDA and transmission assessment surveys) in 11 lymphatic filariasis endemic provinces [5], Thailand attained the WHO criteria for the elimination of lymphatic filariasis as a public health burden in 2017. Recent post-validation surveillance showed transmission limited to a single province, Narathiwat, where microfilaria prevalence remains below the WHO’s provisional transmission threshold of 1% [4]. Sporadic cases and clusters were reported, whereby some cases were associated with cross-border migrations, and concerns of benzimidazole-resistant *W. bancrofti* population were reported [3]. These findings highlight the importance of continuing vigilant disease surveillance both in previously implemented units within lymphatic-filariasis-endemic provinces and among units monitoring migrant populations or populations within lymphatic filariasis receptive areas.

Some potential limitations associated with this report can be declared. First, molecular diagnosis was not performed since microfilaria was detected only in fine-needle aspiration cytologic smear samples. Second, the patient’s dog’s blood sample was not obtained, and thus, the following parasites could not be confirmed or ruled out: *Dirofilaria immitis* infection, which causes heartworm disease in animals; *B. malayi*, which causes filariasis in both animals and humans; and emerging *Brugia pahangi*, which was reported to cause zoonotic infection among children in Thailand and adults in Malaysia [18,19]. We, however, report a case of atypical presentation of human filariasis, which caused organ enlargement, and emphasize how enhanced clinical recognition should be confirmed via fine-needle biopsy and histopathology to enable early treatment and prevent unfavorable consequences.

## 4. Conclusions

We reported a case of parotid filariasis confirmed by histopathology and serology from Southern Thailand. The report suggested that lymphatic filariasis remains present within the area despite attaining a microfilaria prevalence level below the WHO’s provisional transmission threshold of 1%. Early clinical recognition and continued vigilance in regard to disease surveillance were emphasized as a means to control transmission.

## Figures and Tables

**Table 1:** Summary of previously reported microfilaria from cytological smears.

Case	Age/Sex	Clinical Diagnosis	Cytological Diagnosis	Nocturnal Blood Smear	Country	References
1	61/F	Cervical polyp	Inflammatory cells with microfilariae	Negative	India	[10]
2	23/F	Multiple nodular goiter	Malignancy with microfilariae	Negative	India	[14]
3	50/M	Pericardial effusion	Malignancy with microfilariae	Negative	India	[14]
4	55/M	Bronchopneumonia, left upper lobe	Inflammatory cells with microfilariae	Not performed	India	[14]
5	29/M	Lymphadenopathy	Microfilarial lymphadenitis	Negative	India	[14]
6	3/F	Lymphadenopathy	Microfilarial lymphadenitis	Not performed	India	[14]
7	21/F	Breast cyst	Microfilarial lymph cyst	Negative	India	[14]
8	45/M	Lymphadenopathy	Metastatic adenocarcinoma with microfilariae	Negative	India	[11]
9	17/F	Abscess of the left arm	Inflammatory cells with microfilariae	Negative	India	[9]
10	72/M	Bone marrow	Microfilaria with adequate myelopoiesis	*W. bancrofti* detected	India	[12]
11	35/F	Thyroid	Microfilaria with undifferentiated carcinoma	Positive for microfilaria	India	[15]
12	25/M	Parotid swelling	Microfilaria with pleomorphic adenoma	Negative	India	[15]
13	52/F	Breast mass	Microfilaria with adenocarcinoma	Positive for microfilaria	India	[15]
14	55/F	Back pain	Microfilaria with adenocarcinoma	Negative	India	[15]
15	40/M	Gallbladder mass	Microfilaria with adenocarcinoma	Negative	India	[15]
16	60/F	Lung mass	Microfilaria with adenocarcinoma	Positive for microfilaria	India	[15]
17	42/M	Neck mass	Microfilaria with metastatic squamous cell carcinoma	Positive for microfilaria	India	[15]
18	60/F	Pleural effusion	Microfilaria with adenocarcinoma	Negative	India	[17]

## Data Availability

Additional data can be obtained from the corresponding author upon reasonable request.

## Abbreviations

The following abbreviations are used in this manuscript:

CT	Computerized tomography
MDA	Mass drug administration
WHO	World Health Organization
